# Influence of Shear Stress, Inflammation and BRD4 Inhibition on Human Endothelial Cells: A Holistic Proteomic Approach

**DOI:** 10.3390/cells11193086

**Published:** 2022-09-30

**Authors:** Johannes Jarausch, Lisa Neuenroth, Reiner Andag, Andreas Leha, Andreas Fischer, Abdul R. Asif, Christof Lenz, Abass Eidizadeh

**Affiliations:** 1 Department of Clinical Chemistry and Interdisciplinary UMG Laboratory, University Medical Center Goettingen, Robert-Koch-Str. 40, 37075 Goettingen, Germany; 2 Division of Nephrology, Department of Medicine, University Hospital of Wuerzburg, Oberduerrbacher Straße 6, Haus A3, 97080 Wuerzburg, Germany; 3 Department of Medical Statistics, University Medical Center Goettingen, Humboldtallee 32, 37073 Goettingen, Germany; 4 DZHK (German Centre for Cardiovascular Research), Partner Site Goettingen, 37075 Goettingen, Germany; 5 Division Vascular Signaling and Cancer, German Cancer Research Center, Im Neuenheimer Feld 280, 69120 Heidelberg, Germany; 6 Bioanalytical Mass Spectrometry Group, Max Planck Institute for Multidisciplinary Sciences, Am Fassberg 11, 37077 Goettingen, Germany

**Keywords:** HUVEC, shear stress, endothelial, proteomic, BRD4, JQ1, DIA-MS, BET Inhibitor, atherosclerosis

## Abstract

Atherosclerosis is an important risk factor in the development of cardiovascular diseases. In addition to increased plasma lipid concentrations, irregular/oscillatory shear stress and inflammatory processes trigger atherosclerosis. Inhibitors of the transcription modulatory bromo- and extra-terminal domain (BET) protein family (BETi) could offer a possible therapeutic approach due to their epigenetic mechanism and anti-inflammatory properties. In this study, the influence of laminar shear stress, inflammation and BETi treatment on human endothelial cells was investigated using global protein expression profiling by ion mobility separation-enhanced data independent acquisition mass spectrometry (IMS-DIA-MS). For this purpose, primary human umbilical cord derived vascular endothelial cells were treated with TNFα to mimic inflammation and exposed to laminar shear stress in the presence or absence of the BRD4 inhibitor JQ1. IMS-DIA-MS detected over 4037 proteins expressed in endothelial cells. Inflammation, shear stress and BETi led to pronounced changes in protein expression patterns with JQ1 having the greatest effect. To our knowledge, this is the first proteomics study on primary endothelial cells, which provides an extensive database for the effects of shear stress, inflammation and BETi on the endothelial proteome.

## 1. Introduction

Cardiovascular disease (CVD) is the leading cause of death in the US and Europe. The genesis and mortality of CVD are influenced by both modifiable and non-modifiable risk factors [1]. Commonly these factors contribute to atherosclerosis, which highly increases the risk for acute coronary syndrome, stroke, and other CVD [2].

The friction grinding force of shear stress (SS), which blood flow imposes on the vessel wall, modulates endothelial structure and function. Disturbance in SS is one of the leading factors in the progression of atherosclerosis [3]. Inflammatory processes are also crucial for atherosclerosis formation [4]. Low-density lipoprotein (LDL), which is an important transport particle for the lipids [5], is taken up by the endothelium, and deposits within the abluminal extracellular matrix [6] can be oxidized. This triggers local inflammation and the release of pro-inflammatory cytokines and the expression of leukocyte adhesion molecules that further stimulate the infiltration by leukocytes [7,8]. Inhibition of atherosclerosis in an early phase or impeding its progress during the chronic inflammatory phase is intriguing and has therefore given rise to the investigation into possible mechanisms for inhibition of the inflammatory process [9].

Bromodomain and extraterminal (BET) proteins have emerged as important players in the epigenetics of inflammation. They interact with the acetylated lysine residues of histones and function as co-activators for histone acetyltransferases [10]. Thus they regulate gene expression and are of central importance to the execution of the inflammation cascade ^11^. Additionally, acetylated lysine-310 on the RelA subunit is specifically recognized by two bromodomains of bromodomain-containing protein 4 (BRD4), which is essential for the recruitment of NF-κB-target genes [11,12]. Furthermore, BRD4 recruits cyclin-dependent kinase 9 (CDK9) to activate the transcriptional activity of NF-κB [11] and thereby probably initiates the production of proteins in response to inflammatory stimuli.

The first large prospective clinical trial of a BET inhibitor (BETi), RVX-208 (apabetalone) on cardiovascular outcomes has been completed [13,14]. While the effect of apabetalone on the plasma of patients in vivo has been investigated using proteomics [15], little is known about the direct effect of BETis on the vascular cells. In preclinical studies, JQ1, another BETi, successfully inhibited pro-inflammatory processes [16,17].

In an attempt to unravel the effects of JQ1 on cultured ECs in an unbiased manner, we, therefore, designed an experimental setting that emulates typical atherosclerosis-related conditions: BRD4 inhibition with or without shear stress, in the presence or absence of a potent inflammatory stimulus. For this purpose, human umbilical cord-derived vascular endothelial cells (HUVECs) were subjected to mechanical shear stress under inflammation induction and with or without BET inhibition, and their protein abundance profiles were monitored by ion mobility separation-enhanced data-independent acquisition mass spectrometry (IMS-DIA-MS).

## 2. Material and Methods

### 2.1. Cell Culture

All experiments were performed on human umbilical cord-derived vascular endothelial cells (HUVECs) (pooled donors cryopreserved; PromoCell, Heidelberg, Germany) in sterile conditions. Cells were grown and maintained with the endothelial cell growth medium kit (PromoCell, Heidelberg, Germany). The cells were seeded in T-75 culture flasks (Greiner Bio-One, Kremsmuenster, Austria) at 37 °C under a 95% humidified atmosphere with 5% CO_2_. The culture medium was replaced with fresh, warm medium every 2 days until the cells reached 80–90% confluency. At passage four or five, cells were seeded in 60mm in diameter Petri dishes (Greiner Bio-One, Kremsmuenster, Austria) in preparation for shear stress treatment.

### 2.2. Shear Stress (SS) Application, Treatment with TNFα and JQ1

The Petri dishes were pre-coated in 5% gelatine (Merck, Darmstadt, Germany) before seeding. The cell medium was spiked with 5% dextran (Merck, Darmstadt, Germany) to enhance viscosity. SS was applied using a custom-made plate and cone viscometer consisting of a cone with an angle of 0.5° rotating on top of a petri dish (Dr. Fritz Faulhaber GmbH & Co. KG, Schönaich, Germany). Laminar SS was applied continuously for 24 h at 25 dyn/cm^2^—as previously described by others [18]. Afterward, the supernatant was removed, and cells were washed in DPBS without Ca^2+^ and Mg^2+^ (AC-BS-0002, Anprotec, Bruckberg, Germany) and then harvested. After centrifugation (10 min; 800 g) the supernatant was removed, and the pellets were frozen at −20 °C. A 100 µM solution of JQ1 (ApexBio, Houston, TX, USA) was added to the culture medium to attain the desired concentration of 500 nM in the medium, according to other studies and own well-established procedures [19,20]. JQ1 was applied for 20 h alone or parallel to the application of shear stress. After 20 h the cells were washed with 37 °C DPBS and then covered with cell medium and possibly treatment with shear stress continued for another four hours. After initial SS-treatment of 20 h, cell medium was exchanged, cells were washed with DPBS, and new cell medium was added. TNFα (Peprotech, Rocky Hill, EastWindsor, NJ, USA) was applied at a concentration of 25 ng/mL for four h alone or parallel to shear stress, according to our preliminary experiments and well-established methods. Afterward, cells were harvested as mentioned above.

### 2.3. Protein Preparation and Peptide Library

Harvested cells were suspended in 50 µL lysis buffer (50 mM Tris*HCl pH 7.5–7.8, 0.2% SDS supplemented with phosphatase (PhosStop 1:20 (*v*/*v*), Roche, Penzberg, Germany) and protease inhibitors (Complete protease inhibitor 1:20 (*v*/*v*), Roche, Penzberg, Germany), and lysed by sonication in a water bath (4 °C, 6 × 5 min intervals). Protein concentrations were estimated by BCA assay (Pierce BCA protein assay kit, Thermo Fisher Scientific, Dreieich, Germany) and low volume UV detection (Nanodrop 2000,). Excess DNA was degraded by Nuclease digestion (Pierce Universal Nuclease for Cell Lysis, 25kU). Following centrifugation (4 °C, 30 min, 17.000× *g*) the supernatants were transferred to fresh tubes, and proteins precipitated by acetone precipitation (acetone/buffer 5:1 (*v*/*v*), −20 °C, 16 h). Protein precipitates were washed twice with 80% aqueous ethanol, then dissolved by heating in 20 µL LDS loading buffer (70 °C, 10 min). After centrifugation, samples were loaded onto a 4–12% NuPAGE Novex Bis-Tris Minigels (Thermo Fisher Scientific, Dreieich, Germany) and run into the gel for 1.5 cm. Following Coomassie staining, the protein areas were cut out, diced, and subjected to reduction with dithiothreitol, alkylation with iodoacetamide, and finally overnight digestion with trypsin. Tryptic peptides were extracted from the gel, the solution dried in a Speedvac, and kept at −20 °C for further analysis [21]. For generation of a peptide library, equal amount of aliquots from each sample were pooled to a total amount of 200 µg and separated into twelve fractions using a basic pH reversed-phase C18 separation on an FPLC system (äkta pure, Cytiva, Freiburg, Germany) and a staggered pooling scheme. All samples were spiked with a synthetic peptide standard used for retention time alignment (iRT Standard, Schlieren, Switzerland).

### 2.4. Proteomic Analysis

Protein digests were analyzed on a nanoflow chromatography system (nanoElute) hyphenated to a hybrid timed ion mobility-quadrupole-time of flight mass spectrometer (timsTOF Pro, Bruker, Bremen, Germany). In brief, 300 ng equivalents of peptides were dissolved in loading buffer (2% acetonitrile, 0.1% trifluoroacetic acid in water), enriched on a reversed-phase C18 trapping column (0.3 cm × 300 µm, Thermo Fisher Scientific, Dreieich, Germany) and separated on a reversed-phase C18 column with an integrated CaptiveSpray Emitter (Aurora 25 cm × 75 µm, IonOpticks, Fitzroy, VIC, Australia) using a 90 min linear gradient of 5–35% acetonitrile/0.1% formic acid (*v*:*v*) at 300 nl min^−1^, and a column temperature of 50 °C. IMS-DDA-MS analysis was performed in PASEF mode with 10 PASEF scans per topN acquisition cycle [22]. Singly charged precursors were excluded by their position in the *m/z*–ion mobility plane, and precursors that reached a target value of 20,000 arbitrary units were dynamically excluded for 0.4 min. The quadrupole isolation width was set to 2 Th for *m/z* < 700 and to 3 Th for *m/z* > 700. TIMS elution voltages were calibrated linearly to obtain the reduced ion mobility coefficients (1/K0) using three Agilent ESI-L Tuning Mix ions (*m/z* 622, 922 and 1222). Two technical replicates per C18 fraction were acquired. IMS-DIA-MS analysis was performed in diaPASEF mode [23] using 32 × 25 Th isolation windows from *m/z* 400 to 1200 to include the 2+/3+/4+ population in the *m/z*–ion mobility plane. The collision energy was ramped linearly as a function of the mobility from 59 eV at 1/K0 = 1.6Vs cm^−2^ to 20 eV at 1/K0 = 0.6Vs cm^−2^. For further statistical analysis, four independent biological replicates per condition were analyzed by two replicate MS injections each, resulting in a total of eight injections per condition. Protein identification was achieved using the Pulsar algorithm in Spectronaut (version 14.2, Biognosys, Schlieren, Switzerland) using default settings. All DIA data were searched against the UniProtKB *Homo sapiens* reference proteome (revision 01–2020) augmented with a set of 51 known common laboratory contaminants at default settings. For quantitation, up to the 6 most abundant fragment ion traces per peptide, and up to the 10 most abundant peptides per protein were integrated and summed up to provide protein area values. Mass and retention time calibration as well as the corresponding extraction tolerances were dynamically determined. Both identification and quantification results were trimmed to a false discovery rate of 1% using a forward-and-reverse decoy database strategy.

### 2.5. Statistics

**Figure 1:**
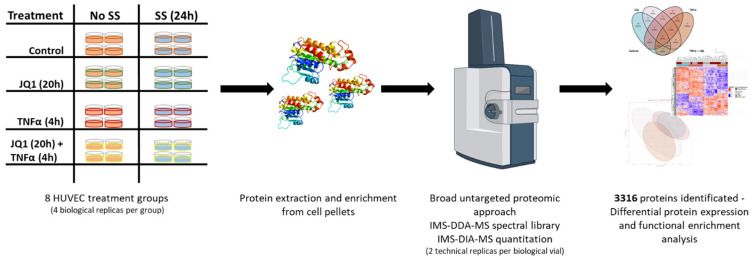
Experimental Design. Eight different treatment groups for human umbilical cord derived vascular endothelial cells (HUVEC) were defined with four biological replicates each. Four groups were subjected to 25 dyn/cm^2^ laminar shear stress (SS) for 24 h. Two were treated as controls, two were exposed to JQ1 (inhibitor of the transcription modulatory bromo- and extra-terminal domain protein BRD4) for the first 20 h, two were exposed to 25 ng/mL TNFα for the last 4 h, two were exposed to 20 h of 500 nM JQ1 and subsequently 4 h of 25 ng/mL TNFα. Proteins were extracted, endoproteolytically digested and analyzed by IMS-DIA-MS, detecting 4037 protein groups. After consistency filtering, 3316 protein groups were submitted to further statistical and functional analysis.

The protein quantifications were log2-transformed, filtered for missing values (*n* > 4 in each condition), quantile normalized between all samples and imputed. Principal component analyses (PCAs) on normalized protein expression values were performed on the full data as well as in subsets with and without SS. Using the R package limma (version 3.42.2) protein expression was modelled using multiple linear models by least squares, and the standard errors were moderated towards a common value using empirical Bayes [24] accounting for the technical replication [25]. As fixed factor, the condition (SS_Control, SS_TNFα, SS_JQ1, SS_TNFα + JQ1, noSS_Control, noSS_TNFα, noSS_JQ1, noSS_TNFα + JQ1) was part of the model. Results from ANOVA-type tests across all groups are presented along results from pairwise contrast tests. The results were reported in gene tables with effect size and significance annotation. Resulting *p* values were adjusted for multiple testing using Benjamini–Hochberg (BH) to control the FDR. The expression levels of significantly differentially expressed proteins were visualized in heatmaps with samples and proteins hierarchically clustered using complete linkage. Expression of top differential proteins was additionally visualized in boxplots. The results from the pairwise contrast tests were also visualized in volcano plots. Functional enrichment towards GO (Gene Ontology) terms and KEGG (Kyoto Encyclopedia of Genes and Genomes) pathways was tested for using enrichment and overrepresentation tests utilizing clusterProfiler (version 3.14.3) [26]. The resulting *p* values have been adjusted for multiple testing using BH to control the FDR. Gene set variation analysis (GSVA) was performed using GSVA (version 1.34.0) to generate expression scores at the gene set levels [27]. The significance level was set to alpha = 5% for all statistical tests. All analyses were performed with the statistic software R (version 3.6.2; R Core Team 2018). ComplexHeatmap (version 2.2.0) was used to generate the heatmaps [28]. During processing of the samples, one sample out of the group treated with SS + JQ1 + TNFα was lost due to a damaged vial, while two had to be excluded from analysis as statistical outliers, one from the group treated only with JQ1 + TNFα and one from the group treated only with SS (Figure 1). Due to the fact that each lost sample was from a different group, the statistical resilience of the remaining samples was still strong; therefore, we proceeded with the evaluation of the remaining 29 samples.

### 2.6. Data Availability

The mass spectrometry proteomics data have been deposited to the ProteomeXchange Consortium via the PRIDE partner repository with the dataset identifier PXD035842.

## 3. Results

### 3.1. Experimental Design

To determine the effect of the BETi JQ1 on primary human endothelial cells in a cellular model, mimicking aspects of atherosclerosis, HUVECs were exposed to laminar SS (25 dyn/cm^2^) with or without 25 ng/mL TNFα treatment in the presence or absence of 500 nM JQ1 and compared with a control group without any treatment. In summary, this resulted in eight treatment groups (Figure 1).

### 3.2. Comprising all Experimental Settings

**Figure 2:**
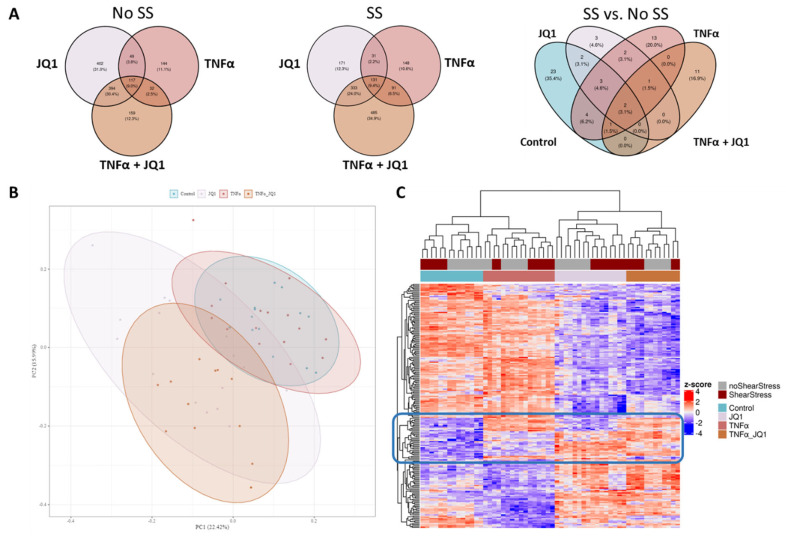
Venn diagrams, principal component analysis and a heatmap of discovered statistically significant differential proteins comparing groups. (**A**) Treatment groups were compared to their respective control with or without shear stress (SS), numbers of distinctive or overlapping significantly differential proteins are annotated. A comparison of all treatment groups with SS compared to all groups without SS was also performed, numbers of distinctive or overlapping significantly differential proteins are annotated. (**B**) Principal component analysis (PCA) of all treatment groups was performed. The first 2 principal components are shown. The treatment groups show distinct clustering, largely dependent on the application of JQ1. (**C**) Heat map of the first 200 differentially expressed proteins discovered by ANOVA-like testing over all treatment groups. The treatment groups separate distinctively according to the received treatment. While JQ1 has the greatest effect, some proteins are strongly affected by the other treatment modalities as well. The blue box marks a group of proteins that separate well between all treatment groups.

As a first step, we performed in-depth qualitative analysis of a pooled reference sample by IMS-DDA-MS following pre-fractionation by basic pH reversed phase C18 separation. We detected 6429 protein groups evidenced by 54,432 peptide sequences at a False Discovery Rate of 1%, which were transcribed into an annotated MS/MS spectral library. Using this library, 4037 proteins groups supported by 34,232 peptides were detected and quantified in the individual samples by single-shot IMS-DIA-MS. After consistency filtering and imputation of missing values, a quantitation matrix containing values for 3.316 proteins was generated and used for statistical analysis. A principal component analysis (PCA) across all treatment groups was performed to assess whether data variation differs from biological grouping. The treatment groups showed distinct clustering dependent on the application of JQ1 (Figure 2B). When comparing the different treatment groups with or without SS to their respective control, the overlapping and distinctive differential proteins could be discovered. Under SS, 1390 significantly differential proteins could be found in comparison with control, while under no SS there were 1297 significant differentially expressed proteins in comparison to control encompassing the different treatment groups. Furthermore, the effect of SS was assessed by comparing the different treatment groups with SS to the groups without SS (Figure 2A). These Venn diagrams show the small effect of SS when compared to the effect of TNFα and JQ1 on the translational profile of HUVECs.

**Figure 3:**
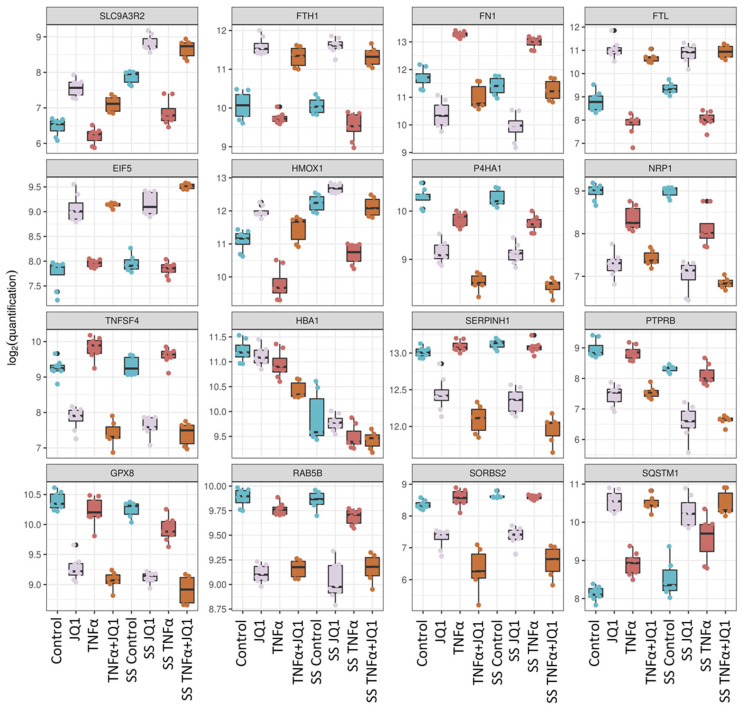
Top 16 significantly differentially expressed proteins between all treatment groups. For each of the proteins the boxplots show protein expression on a log2−scale across all treatment groups. ANOVA-like testing was performed. Na^+^/H^+^ exchange regulatory cofactor 2 (SLC9A3R2), ferritin heavy chain (FTH1), fibronectin (FN1), ferritin light chain (FTL), eukaryotic translation initiation factor 5 (EIF5), heme oxygenase 1 (HMOX1), prolyl 4-hydroxylase subunit alpha-1 (P4HA1), neuropilin-1 (NRP1), tumor necrosis factor ligand superfamily member 4 (TNFSF4), hemoglobin subunit alpha (HBA1), serpin H1 (SERPINH1), receptor-type tyrosine-protein phosphatase beta (PTPRB), probable glutathione peroxidase 8 (GPX8), Ras-related protein Rab-5B (RAB5B), sorbin and SH3 domain-containing protein 2 (SORBS2), sequestosome-1 (SQSTM1).

Of the 1815 differentially expressed proteins across all conditions, the top 200 were analyzed by unbiased hierarchical clustering (Figure 2C), indicating consistent protein expression changes directed by JQ1 and TNFα application rather than by the effect of shear stress. The grouping according to treatment is better captured than the grouping according to shear stress. The top 16 differentially expressed proteins by ANOVA are showing the degree of expression relative to the other treatment groups (Figure 3). For most of those, protein expression seems to be determined by treatment with JQ1; however, for fibronectin 1 (FN1) expression seems to be determined by the application of TNFα, for heme oxygenase 1 (HMOX1) the combination of SS and the application of TNFα seems to affect expression significantly. The expression of sodium/hydrogen exchanger 3 regulator 2 (SLC9A3R2) was greatly affected by the application of SS, just as is the case with hemoglobin subunit alpha (HBA1). Interestingly, while the application of JQ1 seems to hide the effects of SS on sequestosome−1 (SQSTM1), the application of SS by itself appears to enhance the expression of the protein as well. The top 20 differentially expressed proteins discovered by ANOVA are also shown by annotation (Supplementary Table S1).

### 3.3. Proteomic Changes after Shear Stress

**Figure 4:**
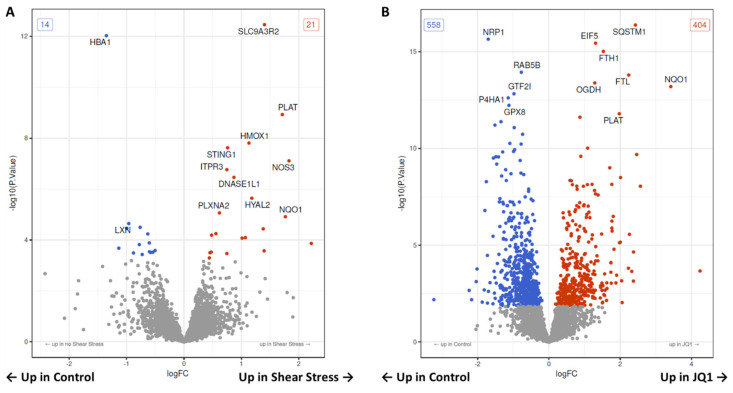
Volcano plot of all tested proteins under SS or JQ1. (**A**) The volcano plot shows for all proteins the results of the contrast test between control with and without shear stress (SS). The x-axis shows the effect size (as log2 fold change), the y-axis shows significance (as −log10 (*p*−value)). The significant differentially expressed proteins are marked by color. Red proteins are upregulated in SS and blue proteins are downregulated. (**B**) The volcano plot shows the differential proteins in the contrast test between control and the JQ1 treatment group without SS. The significant differentially expressed proteins are marked by color. Red proteins are upregulated in JQ1, and blue proteins are downregulated.

By comparing the groups “noSS_Control” and “SS_Control” the proteomic analysis revealed 35 proteins were expressed differently. A total of 21 proteins were significantly upregulated in SS and 14 were significantly downregulated when compared to control (Figure 4A, Supplementary Table S2, and Supplementary Figures S1 and S2A). The top 20 differentially expressed proteins are shown by annotation (Supplementary Table S5). Notably, the aforementioned HMOX1 and SLC9A3R2 are among the most significantly upregulated proteins, while HBA1 is among the most significantly downregulated. Interestingly, nitric oxide synthase 3 (NOS3) and stimulator of interferon response cGAMP interactor 1 (STING1) as well as tissue-type plasminogen activator (PLAT) are among the most significantly upregulated proteins. An overrepresentation analysis referencing the Kyoto Encyclopedia of Genes and Genomes (KEGG) database revealed that four pathways are significantly overrepresented among the differential genes. After adjusting for multiple testing two pathways remain significantly overrepresented. These are “Fluid shear stress and atherosclerosis” and “Complement and coagulation cascades”. The top 20 pathways regardless of significance are shown in an enrichment map (Supplementary Figure S2B). The differential proteins are annotated by color in the most significantly overrepresented pathway “Fluid shear stress and atherosclerosis” (Supplementary Figure S3).

### 3.4. Proteomic Changes after JQ1 Application

**Figure 5:**
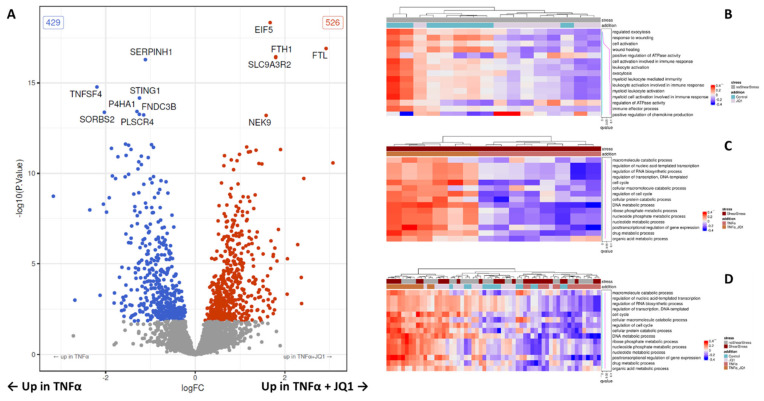
Volcano plot of all tested proteins under JQ1 + TNFα and SS compared to TNFα and SS and heat maps of the results from a gene set variation analysis using GO-terms of the treatment groups. (**A**) The volcano plot shows for all proteins the results of the contrast test between the TNFα treatment group under shear stress (SS) and the TNFα + JQ1 treatment group under SS. The x-axis shows the effect size (as log2 fold change), the y-axis shows significance (as -log10 (*p*-value)). The significant differentially expressed proteins are marked by color. Red proteins are upregulated in TNFα + JQ1 and blue proteins are downregulated. (**B**) The central part of the heat map displays the gene set variation scores of the top 15 overrepresented gene ontology (GO) terms comparing control with JQ1 treatment without SS. The GO terms (rows) are ordered according to significance (the right panel shows *p*-values). The samples (columns) are ordered according to hierarchical clustering using the gene set variation scores. The top panel shows the clustering via a dendrogram, together with sample metadata. The groups separate well. The three significant GO terms “regulated exocytosis”, “response to wounding” and “cell activation” are downregulated under JQ1. (**C**) Comparing TNFα with TNFα + JQ1 under SS the top 15 significantly influenced GO terms are related to cell cycle, transcription, and catabolic state are enriched. The groups separate well. (**D**) Comparing all treatment groups together with and without SS, the enrichment of the respective GO-terms is most pronounced in groups treated with TNFα and JQ1 as well as TNFα irrespective of SS. These groups separate well. The control groups and the groups treated just with JQ1 show less distinctive enrichment.

We have compared HUVECs treated with JQ1 with untreated HUVECSs as a control. The proteomic analysis revealed 558 significantly down- and 404 significantly upregulated proteins after treatment with JQ1, which means 962 were differentially expressed in total (Figure 4B, Supplementary Table S3, and Supplementary Figure S4A). The top 20 significant differentially expressed proteins are shown (Supplementary Table S6). Here, we find proteins such as SQSTM1, neuropilin-1 (NRP1), eucaryotic translation initiation factor 5 (EIF5), ferritin heavy chain (FTH1), and ferritin light chain (FTL), that were already shown to be differentially expressed among the top 16 proteins discovered by ANOVA-like testing over all groups. We carried out an overrepresentation analysis of the differentially expressed gene terms using the Gene Ontology (GO) knowledgebase. Of 4574 tested GO terms, 400 are significantly overrepresented among the higher differential genes. After adjusting for multiple testing 3 GO terms remain significantly overrepresented. We have illustrated the top 15 overrepresented GO terms as a heat map. The GO terms are ordered according to significance. The data suggest that JQ1 treatment downregulates “regulated exocytosis”, “response to wounding” and “cell activation” (Figure 5B). We have illustrated the top 20 overrepresented GO terms in an enrichment map (Supplementary Figure S4B).

### 3.5. Proteomic Changes after JQ1 Application in Shear Stress and Inflammation

Finally, both setups were combined to investigate the effect of JQ1 on HUVECs in our model simulating laminar shear stress in an in vivo vessel, if they were subjected to inflammation. We, therefore, compared HUVECs treated with SS + TNFα to HUVECs treated with SS + TNFα + JQ1. The proteomic analysis revealed 955 proteins to be differentially expressed, 526 were significantly upregulated, and 429 significantly downregulated in the cells treated with JQ1 (Figure 5A and Supplementary Table S4). A heat map depicting the expression of proteins that are differentially expressed in the contrast test is showing distinct clustering (Supplementary Figure S5A). The top 20 differentially expressed proteins discovered are shown by annotation (Supplementary Table S7). It is noteworthy that JQ1 seems to enhance the expression of eukaryotic translation initiation factor 5 (EIF5), and Na^+^/H^+^ exchange regulatory cofactor NHE-RF2 (SLC9A3R2) significantly. Of 3105 tested GO terms, 649 were significantly enriched among the higher differential proteins. After adjusting for multiple testing, 186 GO terms remain significantly enriched. GO terms related to cell cycle, transcription, and metabolic processes are heavily featured among the top 15 enriched GO terms. We have depicted the top 15 enriched GO terms as a heat map restricted to the samples used for this contrast test, as well as a heat map encompassing all treatment groups (Figure 5C,D). JQ1 appears to enhance the enrichment of several GO-Terms relating to transcription and cell cycle, including “regulation of nucleic acid-templated transcription”, “regulation of RNA biosynthetic process”, “regulation of transcription, DNA-templated”, “cell cycle” and “regulation of cell cycle”, furthermore it seems to influence pathways related to the catabolic state, including “macromolecule catabolic process”, “cellular macromolecule catabolic process” and “cellular protein catabolic process” (Supplementary Figure S5B). JQ1 seems to enhance anti-inflammatory translational profiles. This is supported by the overexpression of the ferritin chains “ferritin light chain” (FTL) and “ferritin heavy chain” (FTH), the downregulation of “Tumor necrosis factor ligand superfamily member 4” (TNFSF4), the “stimulator of interferon genes protein” (STING1) and “phospholipid scramblase 4” (PLSCR4), as well as the changes in GO Terms relevant to transcription mentioned above.

## 4. Discussion

This study is, to our knowledge, the first unbiased broad untargeted proteomic approach to investigate the effects of inflammation, shear stress, and BETi treatment on human endothelial cells. We have identified 4037 proteins and could integrate 3316 proteins in our differential statistical analysis. The statistical basis for our study is therefore resilient. Apart from an overlook comprising all treatment groups, it was our aim to investigate the effects of different individual treatments on HUVECs.

### 4.1. Comprising all Treatment Groups

When examining the top 200 differentially expressed proteins over all treatment groups (Figure 2C), it is obvious that the treatment with JQ1 is the primary dividing factor. While the application or lack thereof of TNFα still leads to distinct clustering, the effect of SS over all groups is less pronounced. This is supported by research conducted on several other cell lines in which the transcriptomic and proteomic effect of JQ1 was strongly pronounced, even though results do not lend themselves to direct comparison [29,30,31]. Of the 16 most differentially expressed proteins, only FN1 is largely differentiated by TNFα from the other treatment groups. This verifies our results, as inducibility of fibronectin by TNFα is well documented [32]. Fibronectin can expand the extracellular matrix (ECM) and adversely affect endothelial cells, thus contributing to the infiltration of inflammatory cells and plaque progression [33]. It is therefore noteworthy, that the application of JQ1 seems to largely mitigate the overexpression of FN1 induced by TNFα in HUVECs, potentially inhibiting its atherogenic effects. This correlates well with studies on myofibroblasts, HK-2 cells, and renal tissue, showing that JQ1 does inhibit FN expression in these cell lines [34,35].

### 4.2. The Effect of SS on HUVECs Proteome

SS is defined by the frictional drag to which the arterial wall is exposed and expressed in force/area and is proportional to the product of the blood viscosity and the spatial gradient of the blood velocity in the vessel [36]. Although data differs, it is however accepted that shear stress in the arterial system may reach up to values of about 40 dyn/cm^2^ or even higher [36,37]. SS is highly relevant to the pathophysiology of atherosclerosis, because it is not only able to modulate gene expression through mechanotransduction and shear stress response promotor elements (SSREs), but also because it is capable of transforming the underlying structure of the endothelial cell [38]. When comparing the most differential proteins between SS and control, the most differentially expressed proteins found are SLC9A3R2, HBA1, and HMOX1. SLC9A3R2 is a scaffold protein linking plasma membrane proteins to the actin cytoskeleton and is necessary for phosphorylation and inhibition of SLC9A3, which is a Na^+^/H^+^−exchanger [39]. It has been described as an endothelial marker protein before, however, a SS response has so far not been described [40]. It is associated with a protective role against hypertension, implicating its enhanced expression by SS as a necessary physiological response to arterial strain [41]. As to the SS-dependent suppression of HBA1 translation, we are so far unaware of any studies connecting HBA1 expression and SS in endothelial cells. The significant overexpression of NOS3 validates our experimental setup, as NOS3 is an endothelial household gene, well known to be inducible by exposition to SS [42]. Upregulation of PLAT in the transcriptional profile of ECs by SS has previously been reported [43]. As SS may cause platelet activation by ECs [44], observed overexpression of PLAT may counteract such a thrombogenic mechanism. Furthermore, PLAT is involved in the remodeling of the extracellular matrix and in angiogenesis and is therefore necessary for HUVECs to align parallel to the flow profile [45]. STING1 has been described as a protein involved in immune response and coagulation initiation as well as cell death and apoptosis [46,47] Its overexpression is intriguing as it has previously to our knowledge not been reported in ECs exposed to SS. The two significantly overrepresented pathways, “fluid shear stress and atherosclerosis” and “complement and coagulation cascades”, support the scientific consensus that laminar flow is atheroprotective. This is unsurprising as NOS3, featured in the first pathway, is supposed to have an antithrombotic effect [48] and PLAT features in both pathways, potentially inhibiting an overly coagulatory state as well. Notably, out of the latter pathway, the expression of coagulation factor V (F5) is downregulated under SS, which has to our knowledge not yet been reported. It possesses an important dual role in the coagulation cascade and its downregulation may well support the anti-coagulation profile of SS [49].

### 4.3. The effect of JQ1 on the Endothelial Proteome

When examining the most significant differential proteins under treatment with JQ1, SQSTM1 stands out. Interestingly, the expression of SQSTM1, an autophagy receptor required for selective autophagy of polyubiquitinated cargo [50], seems to be enhanced in HUVECs by the application of laminar flow. This has previously been reported in HUVECs [51]. Paradoxically, recent studies have revealed that laminar SS enhances the process of autophagy, where SQSTM1 seems to be downregulated in arterial endothelial cells by SS [52]. This may well be a result of cell type. Nevertheless, SQSTM1 and autophagy seem to be regulated in response to SS. However, treatment of HUVECs with JQ1 resulted in enhanced expression of SQSTM1 irrespective of SS. Downregulation of SQSTM1-expression has been reported in JQ1-resistant cancer cell lines [53,54]. It may well be the case that the enhancement of transcription is the physiological response, but it could also be that this is a cell type specific phenomenon as in the case of SS. Neuropilin-1 (NRP1) is a cell surface receptor which is important in cancer progression, angiogenesis, immune function and axonal guidance [55]. Suppression of NRP1 expression by JQ1 has already been shown in breast cancer cells [56]. We have now shown the same for HUVECs in a vascular model. Eucaryotic translation initiation factor 5 (EIF5), also affected significantly by JQ1, is a GTPase activating protein, that promotes hydrolysis of GTP. Ferritin heavy chain (FTH1) and ferritin light chain (FTL) two major components of Ferritin are upregulated in response to treatment with JQ1. While this has not been observed for FTL yet, it has been reported, that co-targeting of BRD4 and RAC1 disrupts the MYC/G9a axis and enhances the expression of FTH1 in breast cancer cells [57].

### 4.4. The effect of JQ1 on Inflammation in an In Vitro Model of Atherosclerosis

Among the most significant differentially expressed proteins between the treatment groups SS with TNFα to SS with TNFα and JQ1, we find the aforementioned EIF5, FTH, and FTL. Additionally, serine protease inhibitor 1 (SerpinH1) was suppressed. It is a collagen chaperone taking part in the biosynthesis of collagen, that has been implicated in the pathogenesis of fibrosis, an important part of atherosclerosis [58]. Its overexpression increased cell size, stress fiber formation, mesenchymal and senescence-associated gene expression in human cardiac endothelial cells, while it weakened cell–cell junctions [59]. This implicates it as a therapeutic target in endothelial cells, and our results show, that its suppression by JQ1 may contribute to the atheroprotective potential of BETis. P4HA1 has been shown to be downregulated after treatment with JQ1 when comparing cells solely treated with JQ1 with control. TNFα seems to add to the suppression as well. The suppressive effect of TNFα on P4HA1 expression has previously been described, explaining part of the stronger effect of the combined treatment in our case [60] which demand careful consideration regarding the application of JQ1. Extracellular matrix dysregulation may result in plaque rupture [61], making the positive effects of BETi treatment controversial. SORBS2 is an adapter protein that plays part in assembling signaling complexes and is closely related to cell adhesion [62]. Silencing SORBS2 suppressed fibrosis and proliferation among human glomerular mesangial cells (HGMCs) and human renal glomerular endothelial cells (HRGECs) under high glucose conditions [63]. The observed suppression of SORBS2 under JQ1 and inflammation may therefore in part explain why patients with impaired kidney function did benefit from BETi treatment in the BETonMACE trial on additional subgroup analysis [14].

Our results show that BETis can significantly upregulate the expression of proteins such as proliferating cell nuclear antigen (PCNA) in HUVECs, which is involved in the control of the transcriptional process and is important to DNA damage response [64]. Similarly, high mobility group protein B1 (HMGB1) is upregulated in our study by JQ1 which is known to play an important role in cell growth, proliferation, and death as well autophagy and secretion. Furthermore, it is involved in the recruitment of immune response and inflammation and could help sustain a long-term inflammatory state under stress [65]. One should therefore consider the enrichment of catabolic GO terms as a potentially negative result of JQ1 treatment that warrants further investigation.

### 4.5. Limitations of the Study

As we have studied HUVECs in vitro, results may be particular to that, as it cannot provide the same microenvironment an in vivo setting might offer. It should be noted that we only used a cell model with HUVEC. Atherosclerosis is a complex process that is subject to various influencing factors and involves several vascular as well as non-vascular cells. Our study, on the other hand, focuses on only one vascular cell type, so further similar studies would be useful. Furthermore, an arterial cell model would be useful in further studies. JQ1 is not very specific to a particular BRD. It is highly complementary to BRD2, BRD3, and BRD4 and is considered a pan-BET inhibitor [17]. BET proteins are implicated to promote the expression of cell cycle genes and as corepressors for differentiation markers, as well as in cell cycle arrest and subsequent apoptosis promotion in cancer cells [66]. Concerning SS, one should consider performing the experiments with different strengths and modes such as oscillating and pulsatile SS to investigate the ECs in settings approximating different sections of the vessel tree [36]. While laminar SS is a common stimulus in vitro EC models, and the study of its effects is relevant to large parts of the vessel tree, we concede, that the study of the proteome of ECs under turbulent (oscillating) SS is also highly relevant and requires further research, especially since turbulent/oscillating shear stress also has an inherently atherogenic character. Pathway analyzes should also be performed under these conditions. Furthermore, we did not perform functional testing to assess the outcome of JQ1 treatment in in vivo settings; we limited our study to the effects of JQ1 on the proteomic level. The assessment of in vivo effects requires a translational approach. However, our study was performed on human cells; therefore, a measure of comparability to the effects on human cells in a clinical setting can be assumed.

## 5. Conclusions

In summary, to our knowledge, our study is the first to investigate the influence of shear stress, inflammation, and BET inhibition on human endothelial cells in a very broad untargeted proteomic approach. Using IMS-DIA-MS, we were able to detect and quantify 4037 proteins across all samples. We were also able to identify significantly altered proteins for different treatment groups and show possible induced or suppressed signaling pathways. For the first time globally, unbiased proteome data were generated in HUVEC cells on the influence of (i) shear stress on HUVEC (ii) BET inhibition using JQ1 on HUVEC (iii) JQ1 treatment under shear stress and inflammation on HUVEC. These data can form the basis for identifying possible candidates for diagnostic or prognostic biomarkers or for possible therapeutic approaches in the treatment of atherosclerosis and/or for understanding the pathophysiology of shear stress and JQ1 treatment on endothelial cells. However, considering the complexity of the atherosclerotic process, further studies using other cell types and in vivo models are needed to validate the data found here. Finally, we were able to show the potentially harmful effects of JQ1, which in turn necessitates further, more detailed research into the influence of BETi therapy on cardiovascular diseases.

## Data Availability

The mass spectrometry proteomics data have been deposited to the ProteomeXchange Consortium via the PRIDE partner repository with the dataset identifier PXD035842.

## Supplementary Materials

The following supporting information can be downloaded at: https://www.mdpi.com/article/10.3390/cells11193086/s1, Figure S1: Principal component analysis of all groups under and without SS. Figure S2: Heat map of all tested proteins between control groups under SS vs. no SS and overrepresented KEGG-pathways. Figure S3: Proteins in the overrepresented KEGG-pathway “Fluid shear stress and atherosclerosis” coloured by log fold change from the comparison between shear stress and control groups. Figure S4: Heat map of all tested proteins under JQ1 without SS in comparison to control and enrichment network-map of the top 20 overrepresented GO terms. Figure S5: Heat map of all tested proteins in the TNFα + JQ1 under SS vs. TNFα under SS treatment group and the top 20 enriched GO terms. Table S1: Top 20 significantly differentially expressed proteins discovered among all treatment groups by ANOVA-like testing ordered according to significance. Table S2: Significantly up- or downregulated proteins under shear stress vs. control in relation to all discovered proteins. Table S3: Significantly up- or downregulated proteins in the JQ1 without shear stress group vs. control in relation to all discovered proteins. Table S4: Significantly up- or downregulated proteins in the JQ1 + TNFα with shear stress group vs. TNFα with SS in relation to all discovered proteins. Table S5: Top 20 significantly differentially expressed proteins discovered in the shear stress vs. control group ordered according to significance. Table S6: Top 20 significantly differentially expressed proteins discovered in the JQ1 without shear stress vs. control group ordered according to significance. Table S7: Top 20 significantly differentially expressed proteins discovered in the JQ1 + TNFα under shear stress SS vs. TNFα under shear stress group ordered according to significance.
