# Supplementary material for: Influence of Shear Stress, Inflammation and BRD4 Inhibition on Human Endothelial Cells: A Holistic Proteomic Approach
Source: Cells. 2022 Sep 30;11(19):3086. doi: 10.3390/cells11193086 (PMC9563250; doi:10.3390/cells11193086)
Supplement: Supplementary file 1 [file cells-11-03086-s001.zip › cells-1914399-supplementary.pdf]

Supplement Materials

„Influence of Shear Stress, Inflammation and BRD4 Inhibition on Human Endothelial Cells: A Holistic Proteomic Approach“

Jarausch et al.

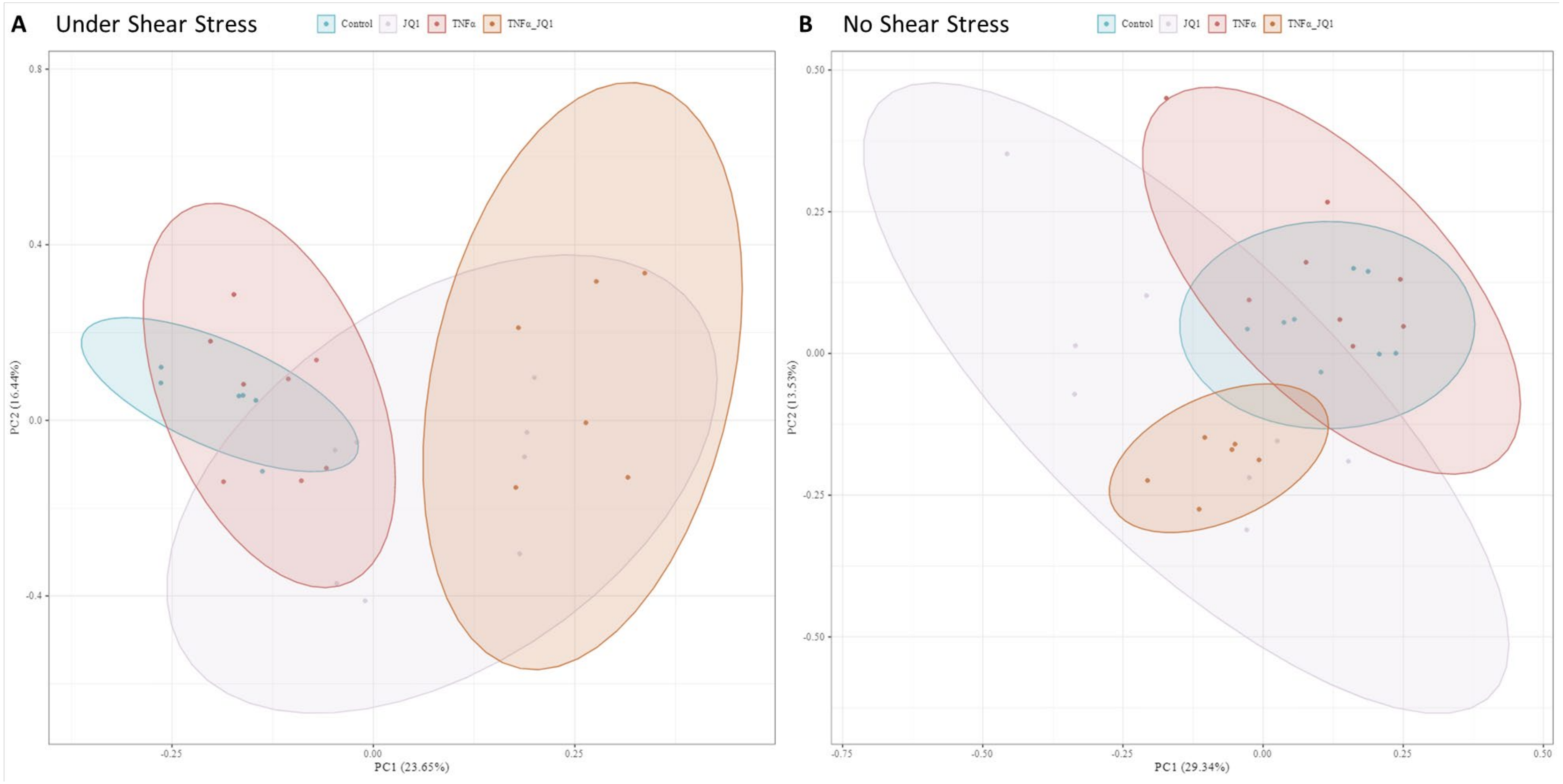

Figure S1. Principal component analysis of all groups under and without SS. (**A**) A PCA of all groups under and without shear stress (SS) was performed. The first two principal components are shown. The groups treated with TNFα + JQ1 show distinct clustering, whereas the groups treated

only with JQ1 did not separate as well. (**B**) Under no SS all groups treated with JQ1 show a pattern distinctive from groups that did not receive JQ1 treatment.

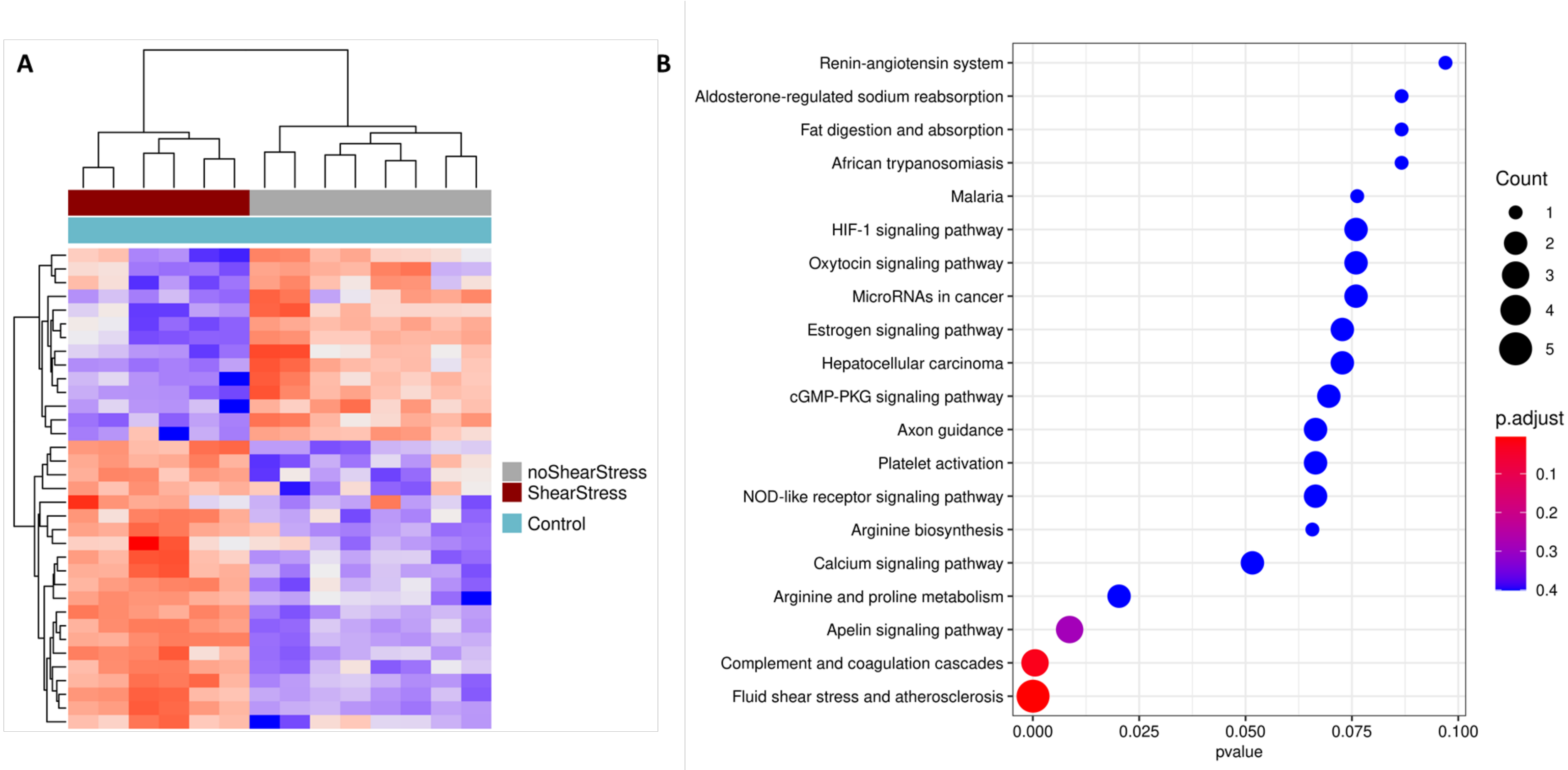

Figure S2. Heat map of all tested proteins between control groups under SS vs. no SS and overrepresented KEGG-pathways. (**A**) Heat map of all proteins between control group under shear stress (SS) or not were selected. The colours show normalized expression values for the differentially expressed proteins (rows) across samples (columns). Both rows and columns are ordered by hierarchical clustering. The clustering is visualized using dendrograms at the top and at the left. The treatment groups show distinct clustering. (**B**) Overrepresented Kyoto Encyclopedia of Genes and Genomes (KEGG) pathways are ordered according to significance (most significantly enriched pathway at the bottom) and coloured by p value. The size of the points represents the count of proteins per pathway. The significantly differential pathways "Fluid shear stress and atherosclerosis" and "Complement and coagulation cascades" are represented at the bottom.

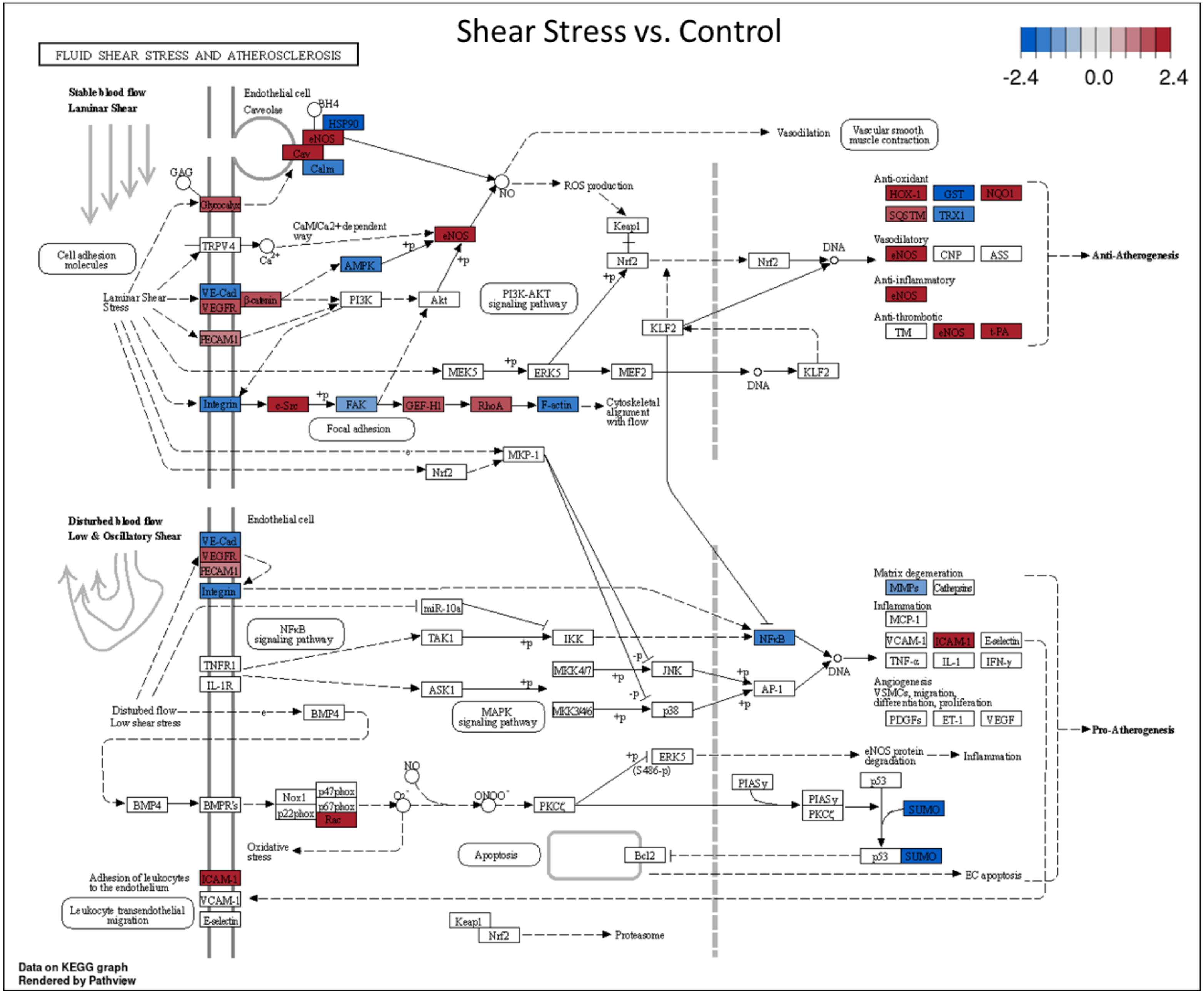

Shear Stress vs. Control
FLUID SHEAR STRESS AND ATHEROSCLEROSIS
-2.4
0.0
2.4
Stable blood flow
Laminar Shear
Endothelial cell
Caveolae
BH4
HSP90
eNOS
Cav
Calm
GAG
Glycocalyx
NO
ROS production
Vasodilation
Vascular smooth muscle contraction
CaM/Ca2+ dependent way
TRPV4
Ca2+
AMPK
Cell adhesion molecules
Laminar Shear Stress
VE-Cad
VEGFR
β-catenin
PI3K
Akt
PI3K-AKT signaling pathway
PECAM1
Keap1
Nrf2
KLF2
DNA
MEK5
ERK5
MEF2
Integrin
c-Src
FAK
GEF-H1
RhoA
F-actin
Cytoskeletal alignment with flow
Focal adhesion
MKP-1
Anti-oxidant
HOX-1
GST
NQO1
SQSTM
TRX1
Vasodilatory
CNP
ASS
Anti-inflammatory
Anti-thrombotic
TM
t-PA
Anti-Atherogenesis
Disturbed blood flow
Low & Oscillatory Shear
miR-10a
NFκB signaling pathway
TAK1
IKK
NFκB
TNFR1
IL-1R
ASK1
MKK4/7
JNK
MKK3/4/6
p38
AP-1
MAPK signaling pathway
Disturbed flow
Low shear stress
BMP4
BMPR's
Nox1
p22phox
p47phox
p67phox
Rac
ONOO-
PKCζ
ERK5
(S486-p)
Oxidative stress
Apoptosis
Bcl2
PIASy
p53
SUMO
eNOS protein degradation
Inflammation
EC apoptosis
Matrix degeneration
MMPs
Cathepsins
Inflammation
MCP-1
VCAM-1
ICAM-1
E-selectin
TNF-α
IL-1
IFN-γ
Angiogenesis
VSMCs, migration, differentiation, proliferation
PDGFs
ET-1
VEGF
Pro-Atherogenesis
Adhesion of leukocytes to the endothelium
Leukocyte transendothelial migration
Proteasome
Data on KEGG graph
Rendered by Pathview

Figure S3. Proteins in the overrepresented KEGG-pathway "Fluid shear stress and atherosclerosis" coloured by log fold change from the comparison between shear stress and control groups. All measured proteins mapped to this pathway are coloured by log fold change (blue for downregulated compared to control, red for upregulated).

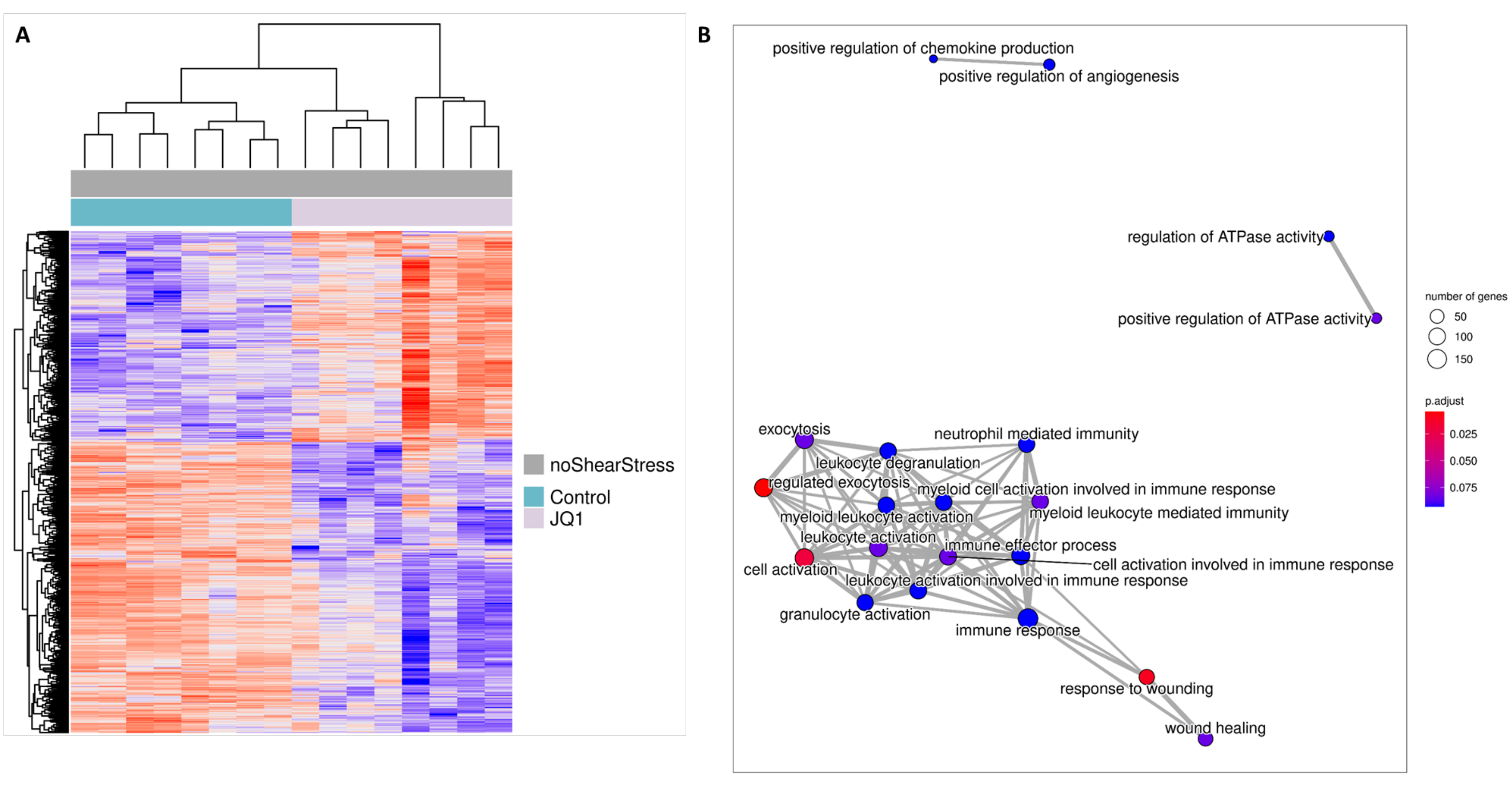

Figure S4. Heat map of all tested proteins under JQ1 without SS in comparison to control and enrichment network-map of the top 20 overrepresented GO terms. (**A**) All proteins between control and JQ1 treatment without shear stress (SS) were selected. The colours show

normalized expression values for the differentially expressed proteins (rows) across samples (columns). Both rows and columns are ordered by hierarchical clustering. The clustering is visualized using dendrograms at the top and at the left. The treatment groups show distinct clustering. (**B**) The dots represent the most overrepresented gene ontology (GO) terms with sizes according to the number of proteins associated with the term. The colour of the dots represents the statistical significance according to the adjusted p value. The width of the connecting lines is according to the number of shared proteins. “Cell activation” and “regulated exocytosis” are closely associated.

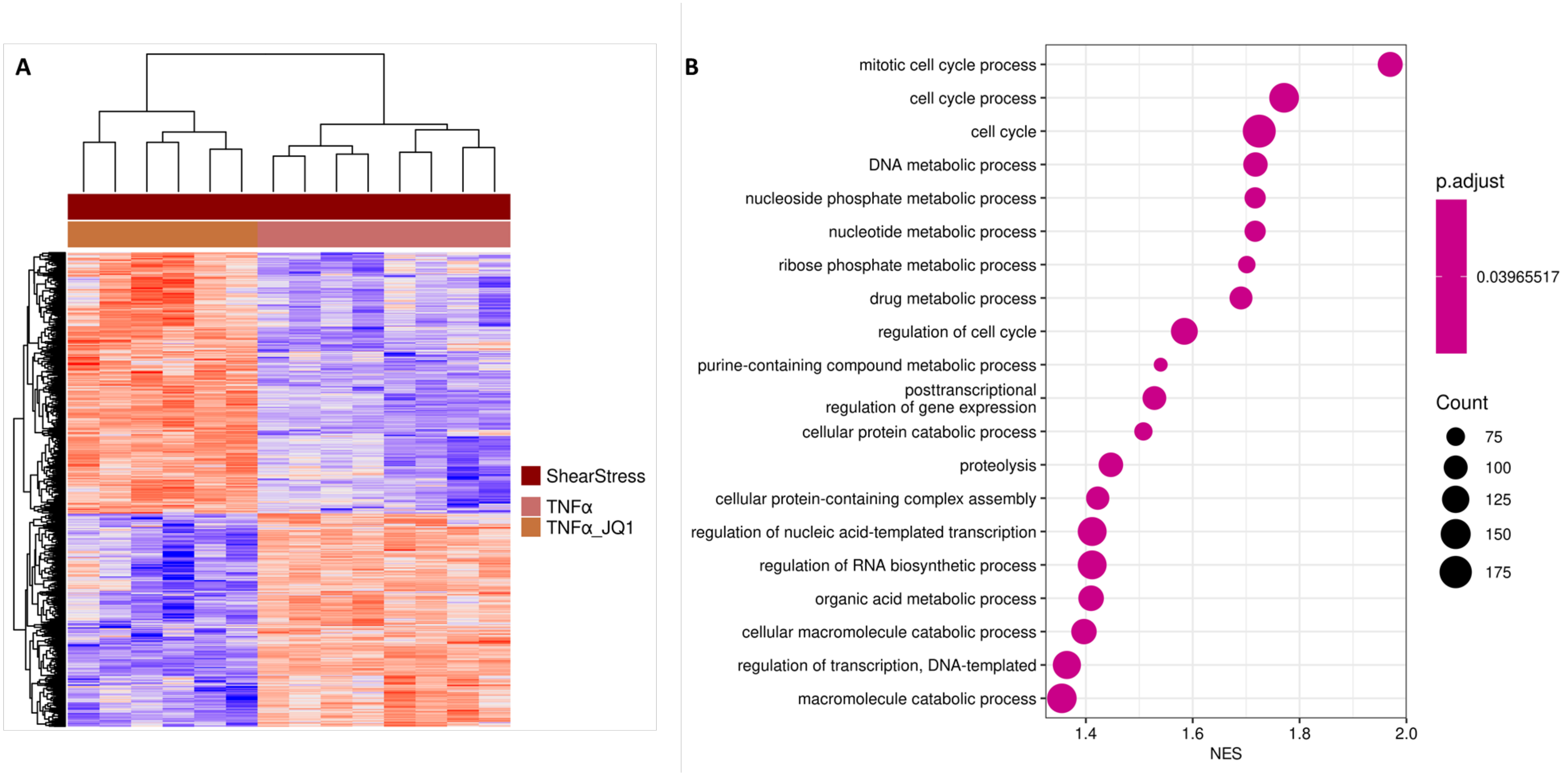

Figure S5. Heat map of all tested proteins in the TNFα + JQ1 under SS vs. TNFα under SS treatment group and the top 20 enriched GO terms. (**A**) Significantly differentially expressed proteins between TNFα and TNFα + JQ1 treatment groups under shear stress (SS). The colours show normalized expression values for the differentially expressed proteins (rows) across samples (columns). Both rows and columns are ordered by

hierarchical clustering. The clustering is visualized using dendrograms at the top and at the left. The treatment groups show distinct clustering. (**B**) The top 20 overrepresented gene ontology (GO) terms comparing TNFα and TNFα + JQ1 treatment groups under SS are ordered according to the normalized enrichment score (most enriched term at the top) and coloured by the adjusted p value. The size of the points represents the size of the term. As can be seen, GO terms related to cell cycle and metabolic processes are most strongly enriched.

Table S1. Top 20 significantly differentially expressed proteins discovered among all treatment groups by ANOVA-like testing ordered according to significance.

Protein	Average Expression	p value	Adjusted p value	Protein Descriptions	Ensgene
**SLC9A3R2**	7.39847331	1.53234E-27	5.08125E-24	Na(+)/H(+) exchange regulatory cofactor NHE-RF2	ENSG00000065054
**FTH1**	10.629059	6.26614E-26	1.03893E-22	Ferritin heavy chain	ENSG00000167996
**FN1**	11.51576524	1.85509E-25	1.56534E-22	Isoform 1 of Fibronectin	ENSG00000115414
**FTL**	9.622760966	1.88822E-25	1.56534E-22	Ferritin light chain	ENSG00000087086
**EIF5**	8.513574672	4.54818E-25	3.01635E-22	Eukaryotic translation initiation factor 5	ENSG00000100664
**HMOX1**	11.46183291	1.96162E-24	1.08412E-21	Heme oxygenase 1	ENSG00000100292
**P4HA1**	9.454305828	2.36744E-23	1.12149E-20	Prolyl 4-hydroxylase subunit alpha-1	ENSG00000122884
**NRP1**	7.887329534	4.82122E-23	1.9984E-20	Neuropilin-1	ENSG00000099250
**TNFSF4**	8.577782052	5.69281E-23	2.09749E-20	Tumor necrosis factor ligand superfamily member 4	ENSG00000117586
**HBA1**	10.32080764	2.76832E-22	9.17974E-20	Hemoglobin subunit alpha	ENSG00000206172
**SERPINH1**	12.67097379	3.97193E-22	1.19736E-19	Serpin H1	ENSG00000149257
**PTPRB**	7.834447345	1.63651E-21	4.52222E-19	Receptor-type tyrosine-protein phosphatase beta	ENSG00000127329
**GPX8**	9.666582483	1.94209E-20	4.83723E-18	Probable glutathione peroxidase 8	ENSG00000164294
**RAB5B**	9.464157828	2.04225E-20	4.83723E-18	Ras-related protein Rab-5B	ENSG00000111540
**SORBS2**	7.765468741	5.81692E-20	1.28593E-17	Sorbin and SH3 domain-containing protein 2	ENSG00000154556

**SQSTM1**	9.594663069	7.12894E-20	1.47747E-17	Sequestosome-1	ENSG00000161011
**CD9**	10.00665303	7.94997E-20	1.55071E-17	Tetraspanin	ENSG00000010278
**CAV1**	11.53978357	2.09811E-19	3.86519E-17	Caveolin-1	ENSG00000105974
**ICAM1**	9.617511569	2.89168E-19	4.98981E-17	Intercellular adhesion molecule 1	ENSG00000090339
**C1QTNF5**	9.101523	3.00953E-19	4.98981E-17	Complement C1q tumor necrosis factor-related protein 5	ENSG00000223953

Table S2. Significantly up- or downregulated proteins under shear stress vs. control in relation to all discovered proteins. 35 proteins are significantly differentially expressed. 21 are up- and 14 are downregulated under shear stress.

Control vs. Shear Stress	Non significant	significant	
Down-Regulated	1553	14	
Up-Regulated	1728	21	
Σ	3281	35	**Total:** 3316

Table S3. Significantly up- or downregulated proteins in the JQ1 without shear stress group vs. control in relation to all discovered proteins.

962 proteins are significantly differentially expressed. 404 are up- and 558 downregulated under JQ1.

Control vs. JQ1	Non significant	significant	
Down-Regulated	1194	558	
Up-Regulated	1160	404	
Σ	2354	962	**Total:** 3316

Table S4. Significantly up- or downregulated proteins in the JQ1 + TNFα with shear stress group vs. TNFα with SS in relation to all discovered proteins. 955 proteins are significantly differentially expressed. 526 are up- and 429 downregulated under JQ1 + TNFα.

TNFα vs. JQ1 + TNFα under Shear Stress	Non significant	significant	
Down-Regulated	1062	429	
Up-Regulated	1299	526	
Σ	2361	955	**Total:** 3316

Table S5. Top 20 significantly differentially expressed proteins discovered in the shear stress vs. control group ordered according to significance.

Protein	Average Expression	p value	Adjusted p value	Protein Descriptions	Ensgene
**SLC9A3R2**	7.39847331	3.47565E-13	1.15253E-09	Na(+)/H(+) exchange regulatory cofactor NHE-RF2	ENSG00000065054
**HBA1**	10.32080764	9.39887E-13	1.55833E-09	Hemoglobin subunit alpha	ENSG00000206172
**PLAT**	7.433489845	1.18065E-09	1.30502E-06	Tissue-type plasminogen activator	ENSG00000104368
**HMOX1**	11.46183291	1.5531E-08	1.28752E-05	Heme oxygenase 1	ENSG00000100292
**STING1**	8.738845293	2.38028E-08	1.5786E-05	Stimulator of interferon response cGAMP interactor 1	ENSG00000184584
**NOS3**	7.073176879	7.74897E-08	4.2826E-05	Nitric oxide synthase. endothelial	ENSG00000164867
**ITPR3**	7.42922369	1.72222E-07	8.15841E-05	Inositol 1,4,5-trisphosphate receptor type 3	ENSG00000096433
**DNASE1L1**	7.406114017	3.46328E-07	0.000143553	Deoxyribonuclease-1-like 1	ENSG00000013563
**HYAL2**	8.708874914	2.28588E-06	0.000842221	Hyaluronidase-2	ENSG00000068001
**PLXNA2**	7.72602269	8.62245E-06	0.002859204	Plexin-A2	ENSG00000076356
**NQO1**	7.474098828	1.21544E-05	0.003663984	NAD(P)H dehydrogenase [quinone] 1	ENSG00000181019
**LXN**	8.313569621	2.2551E-05	0.006231583	Latexin	ENSG00000079257
**TPI1**	11.45701069	3.17862E-05	0.008080394	Triosephosphate isomerase	ENSG00000111669
**F5**	8.466895328	3.48665E-05	0.008080394	Coagulation factor V	ENSG00000198734
**GYG1**	7.465747086	3.65518E-05	0.008080394	Glycogenin-1	ENSG00000163754
**PLEC.Q15149_3**	9.633755621	5.64375E-05	0.011312555	Isoform 3 of Plectin	
**DPYSL2**	10.64734467	5.79956E-05	0.011312555	Dihydropyrimidinase-related protein 2	ENSG00000092964
**PLEC**	11.58752569	6.46601E-05	0.011911834	Plectin	ENSG00000178209
**PLPP3**	8.038891931	8.09454E-05	0.01401305	Phospholipid phosphatase 3	ENSG00000162407
**PODXL**	8.255463603	8.45178E-05	0.01401305	Podocalyxin	ENSG00000128567

Table S6. Top 20 significantly differentially expressed proteins discovered in the JQ1 without shear stress vs. control group ordered according to significance.

Protein	Average Expression	p value	Adjusted p value	Protein Descriptions	Ensgene
**SQSTM1**	9.594663069	4.21271E-17	1.39694E-13	Sequestosome-1	ENSG00000161011
**NRP1**	7.887329534	2.26174E-16	3.74997E-13	Neuropilin;Neuropilin-1	ENSG00000099250
**EIF5**	8.513574672	3.64909E-16	4.03346E-13	Eukaryotic translation initiation factor 5	ENSG00000100664
**FTH1**	10.629059	9.64039E-16	7.99188E-13	Ferritin heavy chain	ENSG00000167996
**RAB5B**	9.464157828	1.15573E-14	7.66481E-12	Ras-related protein Rab-5B	ENSG00000111540
**FTL**	9.622760966	1.61977E-14	8.95191E-12	Ferritin light chain	ENSG00000087086
**OGDH**	9.473688552	4.13929E-14	1.96084E-11	2-oxoglutarate dehydrogenase, mitochondrial	ENSG00000105953
**NQO1**	7.474098828	6.41831E-14	2.66039E-11	NAD(P)H dehydrogenase [quinone] 1	ENSG00000181019
**GTF2I**	8.301597414	1.50104E-13	5.53051E-11	General transcription factor II-I	ENSG00000263001
**P4HA1**	9.454305828	2.44369E-13	8.10329E-11	Prolyl 4-hydroxylase subunit alpha-1	ENSG00000122884
**GPX8**	9.666582483	5.94626E-13	1.79253E-10	Probable glutathione peroxidase 8	ENSG00000164294
**PLAT**	7.433489845	1.61796E-12	4.47096E-10	Tissue-type plasminogen activator	ENSG00000104368
**PI4K2A**	7.497889259	2.43798E-12	6.21872E-10	Phosphatidylinositol 4-kinase type 2-alpha	ENSG00000155252
**A2M**	9.852662879	4.1507E-12	9.83122E-10	Alpha-2-macroglobulin	ENSG00000175899
**PTPRB**	7.834447345	6.24441E-12	1.38043E-09	Receptor-type tyrosine-protein phosphatase beta	ENSG00000127329
**CCAR2**	9.185597207	8.39148E-12	1.73914E-09	Cell cycle and apoptosis regulator protein 2	ENSG00000158941
**AP2M1**	10.23082283	1.85962E-11	3.62736E-09	Isoform 2 of AP-2 complex subunit mu	ENSG00000161203
**DPYSL2**	10.64734467	5.49059E-11	1.01149E-08	Dihydropyrimidinase-related protein 2	ENSG00000092964
**MAGED2**	8.564668431	5.93056E-11	1.03504E-08	Melanoma-associated antigen D2	ENSG00000102316
**SLC9A3R2**	7.39847331	9.63849E-11	1.59806E-08	Na(+)/H(+) exchange regulatory cofactor NHE-RF2	ENSG00000065054

Table S7. Top 20 significantly differentially expressed proteins discovered in the JQ1 + TNFα under shear stress SS vs. TNFα under shear stress group ordered according to significance.

Protein	Average Expression	p value	Adjusted p value	Protein Descriptions	Ensgene
**EIF5**	8.513574672	4.58533E-19	1.5205E-15	Eukaryotic translation initiation factor 5	ENSG00000100664
**FTL**	9.622760966	1.24105E-17	2.05765E-14	Ferritin light chain	ENSG00000087086
**FTH1**	10.629059	3.55049E-17	3.25015E-14	Ferritin heavy chain	ENSG00000167996
**SLC9A3R2**	7.39847331	3.92057E-17	3.25015E-14	Na(+)/H(+) exchange regulatory cofactor NHE-RF2	ENSG00000065054
**SERPINH1**	12.67097379	5.09695E-17	3.3803E-14	Serpin H1	ENSG00000149257
**TNFSF4**	8.577782052	1.66871E-15	9.2224E-13	Tumor necrosis factor ligand superfamily member 4	ENSG00000117586
**STING1**	8.738845293	6.79797E-15	3.2203E-12	Stimulator of interferon response cGAMP interactor 1	ENSG00000184584
**P4HA1**	9.454305828	3.76871E-14	1.53815E-11	Prolyl 4-hydroxylase subunit alpha-1	ENSG00000122884
**SORBS2**	7.765468741	4.1747E-14	1.53815E-11	Sorbin and SH3 domain-containing protein 2	ENSG00000154556
**PLSCR4**	8.406305397	5.40921E-14	1.72575E-11	Phospholipid scramblase 4	ENSG00000114698
**FNDC3B**	8.104490586	5.84412E-14	1.72575E-11	Fibronectin type III domain-containing protein 3B	ENSG00000075420
**NEK9**	6.827641138	6.24516E-14	1.72575E-11	Serine/threonine-protein kinase Nek9	ENSG00000119638
**MYADM**	10.08777569	2.4017E-12	6.11818E-10	Myeloid-associated differentiation marker	ENSG00000179820
**EOGT**	9.176582431	2.59305E-12	6.11818E-10	EGF domain-specific O-linked N-acetylglucosamine transferase	ENSG00000163378
**IGFBP7**	8.813039707	2.76757E-12	6.11818E-10	Insulin-like growth factor-binding protein 7	ENSG00000163453
**PCNA**	9.368632103	3.45943E-12	7.08945E-10	Proliferating cell nuclear antigen	ENSG00000132646
**MOGS**	10.73190186	3.63452E-12	7.08945E-10	Mannosyl-oligosaccharide glucosidase	ENSG00000115275
**CD9**	10.00665303	4.13328E-12	7.61441E-10	Tetraspanin	ENSG00000010278
**RALA**	10.97131834	4.74958E-12	8.00418E-10	Ras-related protein Ral-A	ENSG00000006451
**GCLM**	8.503469345	4.82761E-12	8.00418E-10	Glutamate-cysteine ligase regulatory subunit	ENSG00000023909
